# Higher Particulate Matter Deposition in Alveolar Region Could Accelerate Body Fat Accumulation in Obstructive Sleep Apnea

**DOI:** 10.1021/acsenvironau.2c00034

**Published:** 2022-09-23

**Authors:** Nguyen Thanh Tung, Shang-Yang Lin, Wen-Te Liu, Yi-Chun Kuan, Chih-Da Wu, Huynh Nguyen Xuan Thao, Hoang Ba Dung, Tran Phan Chung Thuy, Hsiao-Chi Chuang

**Affiliations:** † International Ph.D. Program in Medicine, College of Medicine, Taipei Medical University, Taipei 11031, Taiwan; ‡ Otorhinolaryngology Department, Cho Ray Hospital, Ho Chi Minh City 700000, Vietnam; § Sleep Center, Shuang Ho Hospital, Taipei Medical University, New Taipei City 23561, Taiwan; ∥ Taipei Neuroscience Institute, Taipei Medical University, Taipei 11031, Taiwan; ⊥ Department of Neurology, Taipei Medical University Shuang Ho Hospital, New Taipei City 23561, Taiwan; # Department of Neurology, School of Medicine, College of Medicine, Taipei Medical University, Taipei 11031, Taiwan; ∇ Department of Geomatics, National Cheng Kung University, Tainan 70101, Taiwan; ○ National Institute of Environmental Health Sciences, National Health Research Institutes, Miaoli 350, Taiwan; ● Otorhinolaryngology Department, Faculty of Medicine, Vietnam National University Ho Chi Minh City, Ho Chi Minh City 700000, Vietnam; □ School of Respiratory Therapy, College of Medicine, Taipei Medical University, Taipei, 11031, Taiwan; ■ Division of Pulmonary Medicine, Department of Internal Medicine, Shuang Ho Hospital, Taipei Medical University, New Taipei City, 23561, Taiwan; ▲ Cell Physiology and Molecular Image Research Center, Wanfang Hospital, Taipei Medical University, Taipei, 116, Taiwan

**Keywords:** air pollution, fat distribution, lung, MPPD, PM_2.5_, upper airway

## Abstract

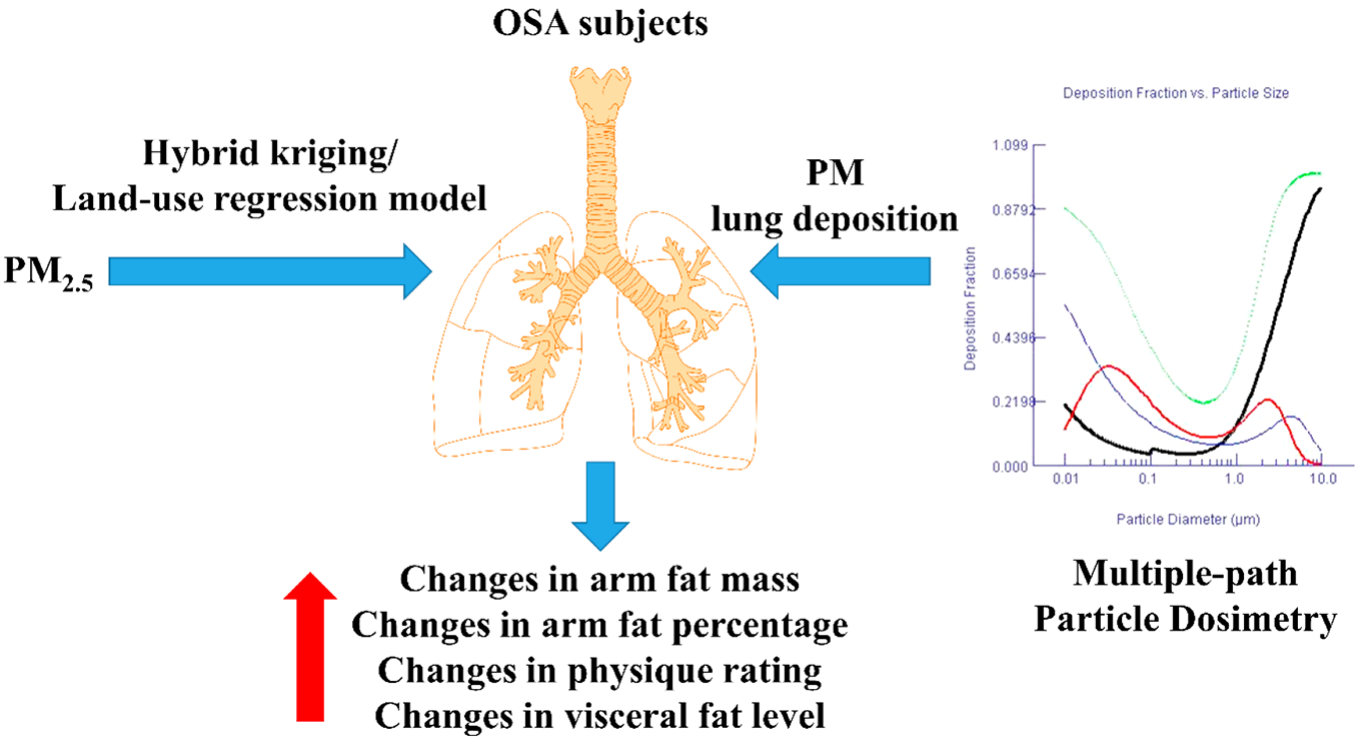

We conducted a cross-sectional study to investigate associations of particulate matter (PM) of less than 2.5 μm in aerodynamic diameter (PM_2.5_) and PM deposition with nocturnal changes in body composition in obstructive sleep apnea (OSA) patients. A bioelectric impedance analysis was used to measure the pre- and postsleep body composition of 185 OSA patients. Annual exposure to PM_2.5_ was estimated by the hybrid kriging/land-use regression model. A multiple-path particle dosimetry model was employed to estimate PM deposition in lung regions. We observed that an increase in the interquartile range (IQR) (1 μg/m^3^) of PM_2.5_ was associated with a 20.1% increase in right arm fat percentage and a 0.012 kg increase in right arm fat mass in OSA (*p* < 0.05). We observed that a 1 μg/m^3^ increase in PM deposition in lung regions (i.e., total lung region, head and nasal region, tracheobronchial region, and alveolar region) was associated with increases in changes of fat percentage and fat mass of the right arm (β coefficient) (*p* < 0.05). The β coefficients decreased as follows: alveolar region > head and nasal region > tracheobronchial region > total lung region (*p* < 0.05). Our findings demonstrated that an increase in PM deposition in lung regions, especially in the alveolar region, could be associated with nocturnal changes in the fat percentage and fat mass of the right arm. PM deposition in the alveolar region could accelerate the body fat accumulation in OSA.

Previous studies have demonstrated the associations between air pollution and body composition, especially fat mass and total muscle mass.^1^ Previous findings showed that each increase of 1.4 μg/m^3^ in particulate matter (PM) of less than 2.5 μm in aerodynamic diameters (PM_2.5_) resulted in a 0.4 kg decrease in total muscle mass and 0.7 kg increase in total fat mass (all *p* < 0.05).^1^ Air pollution exposure was reported to be associated with an increased risk of a sleep breathing disorder such as obstructive sleep apnea (OSA).^2^ PM lung-deposited quantification and its clearance in the respiratory tract is important to assess health risks. Previous studies reported the deposited fraction of inhaled PM in human airways.^3,4^ Previous findings showed that PM_2.5_ deposition was 80% in head and nasal region, 7% in tracheobronchial region, and 13% in the alveolar region.^4^ Nevertheless, associations of PM deposition in lungs after inhalation with changes in the overnight body composition in OSA patients are poorly understood. This cross-sectional study was performed to examine the associations of ambient PM_2.5_ and its deposition in lung regions with nocturnal changes in the body composition of OSA patients.

The Taipei Medical University (Taipei, Taiwan)’s joint institutional review board (TMU-JIRB no. N201912095) approved this study. Inclusion criteria were participants aged 20–80 years with apnea–hypopnea index (AHI) ≥ 5 events per hour recruited from a sleep center in New Taipei City (Taiwan) from 1 January 2019 to 31 December 2019. We excluded subjects with comorbidities such as venous insufficiency, cardiopulmonary diseases, hypertension, heart failure, diabetes mellitus, renal failure, and hemodialysis patients. Subjects who traveled abroad or moved to other regions of Taiwan six months prior to the study were also excluded.

Air pollutant (PM_2.5_, nitrogen dioxide (NO_2_), and ozone (O_3_)) levels were collected from the Taiwan Environmental Protection Administration (EPA)’s air quality monitoring stations to develop the prediction model. The annual exposure to air pollutants of each subject was then estimated by using the hybrid kriging/land-use regression method.^5^ We further applied annual PM_2.5_ levels to calculate the PM deposition in lung regions (i.e., head and nasal region, alveolar region, tracheobronchial region, and total lung region) by using the multiple-path particle dosimetry (MPPD) model (MPPD ver. 3.04 for Windows, Applied Research Associates, Albuquerque, NM, USA).^6^ PM deposition was calculated based on 625 mL of tidal volume, a functional residual capacity of 3300 mL, a respiratory rate of 12 breaths/min, and 50 mL of upper respiratory tract volume.

Polysomnography (PSG) analysis was performed with an Embla N7000 (Medcare, Reykjavik, Iceland) digital system. Sleep parameters collected through overnight PSG included total sleep time, sleep efficiency, wake after sleep onset, arousal index, oxygen saturation, and the AHI. We defined the AHI as the total number of hypopneas and apneas per hour of sleep.

We used a Tanita MC-780 (Tanita, Tokyo, Japan) system to measure presleep and postsleep body composition. We conducted presleep measurement before PSG (21:00–22:00). Subjects were requested to fast for 3 h prior to the procedure, urinate, remove their footwear, stand on the scale platform, and hold the device grips while keeping the arms straight down. Participants were not permitted to eat or drink between the first body composition measurement and the next morning’s measurement. After the subject awakened the next morning (06:00–07:00), we immediately performed the postsleep evaluation. We defined differences (delta; Δ) in body composition pre- and postsleep as the nocturnal changes of these variables. The weight of fat in a body segment was determined as the segmental fat mass. The segmental fat percentage was characterized as the weight of segmental fat as the percentage of total segmental weight. Physique rating was characterized as the ratio of total muscle mass and body fat mass.

To examine the association between an interquartile range (IQR) increase in annual mean PM_2.5_ levels and changes in body composition, a multiple linear regression was performed. These associations were demonstrated as a regression coefficient (β) multiplied by the IQR in annual mean PM_2.5_ concentrations. To examine the associations (β coefficient) of 1 year average PM deposition in lung regions with changes in body composition, multiple linear regression was employed. A multiple linear regression model was adjusted for sex, age, and the body mass index (BMI). In addition, we also used the two-pollutant model to adjust for the potential confounding effects of copollutants, including NO_2_ (Table S1 in the Supporting Information) and O_3_ (Table S2). We conducted statistical analyses by using SPSS Statistics software (SPSS ver. 22.0.0.0 for Windows, Chicago, IL, USA).

As depicted in Table 1, there were 185 OSA subjects in the study. Their mean age was 48.8 ± standard deviation 12.6 years, male subjects represented 74.6%, their average BMI was 27.59 ± 4.48 kg/m^2^, and their mean AHI was 37.55 ± 23.01 events/h.

**Table 1:** Basic Characteristics of Study Subjects^a^

characteristics	mean ± standard deviation (SD)
total	185
age, years	48.8 ± 12.6
male, %	74.6
body-mass index, kg/m^2^	27.59 ± 4.48
sleep parameters	
total sleep time, h	4.7 ± 1.0
sleep efficiency, %	76.34 ± 16.98
WASO, min	67.3 ± 52.9
arousals, events/h	26.00 ± 15.5
mean SpO_2_, %	95.23 ± 2.23
AHI, events/h (min, max)	37.55 ± 23.01 (5.40, 103.40)

a Definitions of abbreviations: AHI, apnea–hypopnea index; SpO_2_, oxygen saturation measured by pulse oximetry; WASO, wake after sleep onset.

We observed that the 1 year mean concentration of PM_2.5_ was 18.43 ± 1.38 μg/m^3^ (Table 2). Meanwhile, the PM concentrations were 17.37 ± 1.30 μg/m^3^ in the total lung region, 6.70 ± 0.50 μg/m^3^ in the head and nasal region, 6.96 ± 0.52 μg/m^3^ in the tracheobronchial region, and 3.70 ± 0.28 μg/m^3^ in the alveolar region.

**Table 2:** Annual Average Concentrations of Nitrogen Dioxide (NO_2_), Ozone (O_3_), Particulate Matter with an Aerodynamic Diameter of Less Than 2.5 μm (PM_2.5_) and Their Depositions in the Total Lung, Head and Nasal, Tracheobronchial, and Alveolar Regions^a^

	concentration
NO_2_, ppb	18.48 ± 3.08
O_3_, ppb	27.89 ± 1.31
PM_2.5_, μg/m^3^	18.43 ± 1.38
respiratory tract region, μg/m^3^	
total lung region	17.37 ± 1.30
head and nasal region	6.70 ± 0.50
tracheobronchial region	6.96 ± 0.52
alveolar region	3.70 ± 0.28

a Definition of abbreviation: ppb, parts per billion.

The association of 1 year average PM_2.5_ concentrations and PM deposition with the overnight body composition changes is demonstrated in Table 3. We observed that an increase in the IQR (1 μg/m^3^) of PM_2.5_ was associated with a 20.1% increase in the right arm fat percentage and a 0.012 kg increase in the right arm fat mass (all *p* < 0.05). We observed that a 1 μg/m^3^ increase of PM_2.5_ was associated with 0.620 point reduction in changes of physique rating (*p* < 0.05). We observed that a 1 μg/m^3^ increase in particle deposition in lung regions (i.e., the total lung region, the head and nasal region, the tracheobronchial region, and the alveolar region) was significantly associated with increases in changes in the right arm fat percentage and right arm fat mass and was associated with decreases in changes in the physique rating (all *p* < 0.05). After adjustment for O_3_ in the two-pollutant model, we found that an increase in the IQR of PM_2.5_ was associated with a 0.071 kg increase in changes of the visceral fat level (*p* < 0.05) (Table S2).

**Table 3:** Associations of an Interquartile Range (IQR) Increase in 1 year Average Concentrations of PM_2.5_ and Associations (β Coefficient) of 1 year Average Concentrations of PM Deposition in Various Lung Regions (i.e., Total Lung, Head and Nasal, Tracheobronchial, and Alveolar Regions) with Changes in Body Composition Parameters in 185 Obstructive Sleep Apnea Patients^a^

			PM deposition in lung regions, β coeff (CI 95%)			
	changes in body composition mean ± SD	1 year PM_2.5_ IQR*β coeff (CI 95%)	total lung	head and nasal	tracheobronchial	alveolar
fat percent, %	–0.60 ± 2.16	0.198 (−0.022, 0.418)	0.210 (−0.023, 0.444)	0.545 (−0.060, 1.150)	0.525 (−0.058, 1.107)	0.986 (−0.108, 2.081)
fat mass, kg	–0.20 ± 1.80	0.163 (−0.020, 0.345)	0.173 (−0.021, 0.367)	0.448 (−0.055, 0.951)	0.431 (−0.053, 0.915)	0.810 (−0.100, 1.720)
muscle mass, kg	0.87 ± 1.69	–0.135 (−0.306, 0.036)	–0.143 (−0.325, 0.038)	–0.372 (−0.842, 0.099)	–0.358 (−0.810, 0.095)	–0.672 (−1.523, 0.179)
visceral fat level, kg	–0.16 ± 0.56	0.049 (−0.010, 0.108)	0.052 (−0.010, 0.115)	0.136 (−0.026, 0.298)	0.131 (−0.025, 0.287)	0.246 (−0.048, 0.540)
bone mass, kg	0.05 ± 0.10	–0.008 (−0.018, 0.002)	–0.008 (−0.019, 0.002)	–0.021 (−0.049, 0.006)	–0.021 (−0.048, 0.006)	–0.039 (−0.089, 0.012)
fat free mass, kg	0.92 ± 1.78	–0.143 (−0.323, 0.037)	–0.152 (−0.343, 0.039)	–0.393 (−0.889, 0.102)	–0.378 (−0.855, 0.098)	–0.711 (−1.608, 0.185)
Body Water						
TBW, kg	1.82 ± 1.90	–0.111 (−0.300, 0.078)	–0.118 (−0.319, 0.083)	–0.306 (−0.826, 0.214)	–0.294 (−0.794, 0.206)	–0.553 (−1.494, 0.387)
ECW, kg	0.31 ± 0.50	–0.032 (−0.083, 0.019)	–0.034 (−0.088, 0.020)	–0.088 (−0.227, 0.051)	–0.085 (−0.219, 0.050)	–0.159 (−0.411, 0.093)
ICW, kg	1.51 ± 1.43	–0.079 (−0.221, 0.062)	–0.084 (−0.234, 0.066)	–0.218 (−0.607, 0.171)	–0.210 (−0.584, 0.165)	–0.394 (−1.098, 0.310)
Metabolism						
BMR, kJ	112.32 ± 204.68	–16.445 (−37.245, 4.355)	–17.458 (−39.538, 4.623)	–45.253 (−102.491, 11.984)	–43.540 (−98.610, 11.531)	–81.857 (−185.391, 21.678)
METAAGE, year	–1.07 ± 3.02	–0.085 (−0.408, 0.238)	–0.090 (−0.433, 0.253)	–0.234 (−1.123, 0.655)	–0.225 (−1.081, 0.630)	–0.424 (−2.032, 1.184)
Right Leg						
FATP, %	–0.97 ± 1.70	0.149 (−0.015, 0.313)	0.158 (−0.016, 0.332)	0.410 (−0.041, 0.860)	0.394 (−0.039, 0.827)	0.741 (−0.074, 1.556)
FATM, kg	0.10 ± 0.42	0.021 (−0.020, 0.062)	0.022 (−0.021, 0.065)	0.057 (−0.055, 0.170)	0.055 (−0.052, 0.163)	0.104 (−0.099, 0.307)
FFM, kg	0.67 ± 0.30	–0.004 (−0.034, 0.025)	–0.004 (−0.036, 0.027)	–0.011 (−0.093, 0.070)	–0.011 (−0.089, 0.067)	–0.021 (−0.168, 0.126)
PMM, kg	0.63 ± 0.29	–0.004 (−0.032, 0.025)	–0.004 (−0.034, 0.026)	–0.010 (−0.089, 0.069)	–0.010 (−0.086, 0.066)	–0.019 (−0.161, 0.124)
IMP, Ω	–26.86 ± 11.52	0.155 (−0.940, 1.250)	0.165 (−0.998, 1.327)	0.427 (−2.586, 3.440)	0.411 (−2.488, 3.310)	0.772 (−4.678, 6.223)
Left Leg						
FATP, %	–1.05 ± 1.87	0.169 (−0.010, 0.349)	0.180 (−0.011, 0.371)	0.466 (−0.029, 0.960)	0.448 (−0.028, 0.924)	0.843 (−0.052, 1.737)
FATM, kg	0.10 ± 0.40	0.025 (−0.013, 0.063)	0.027 (−0.013, 0.067)	0.070 (−0.035, 0.175)	0.067 (−0.034, 0.168)	0.126 (−0.063, 0.316)
FFM, kg	0.68 ± 0.28	0.004 (−0.025, 0.032)	0.004 (−0.026, 0.034)	0.011 (−0.068, 0.089)	0.010 (−0.065, 0.085)	0.019 (−0.122, 0.160)
PMM, kg	0.63 ± 0.27	0.006 (−0.021, 0.033)	0.007 (−0.022, 0.035)	0.017 (−0.057, 0.092)	0.016 (−0.055, 0.088)	0.031 (−0.104, 0.166)
IMP, Ω	–28.54 ± 12.18	–0.137 (−1.300, 1.026)	–0.145 (−1.380, 1.089)	–0.377 (−3.577, 2.824)	–0.362 (−3.442, 2.717)	–0.682 (−6.471, 5.108)
Right Arm						
FATP, %	0.42 ± 1.90	0.201 (0.002, 0.400)*	0.213 (0.002, 0.424)*	0.553 (0.006, 1.100)*	0.532 (0.006, 1.059)*	1.001 (0.011, 1.990)*
FATM, kg	0.04 ± 0.10	0.012 (0.001, 0.023)*	0.013 (0.001, 0.025)*	0.034 (0.004, 0.064)*	0.033 (0.004, 0.061)*	0.061 (0.007, 0.115)*
FFM, kg	0.08 ± 0.13	0.004 (−0.010, 0.018)	0.005 (−0.010, 0.019)	0.012 (−0.026, 0.050)	0.011 (−0.025, 0.048)	0.021 (−0.048, 0.090)
PMM, kg	0.07 ± 0.13	0.004 (−0.010, 0.018)	0.004 (−0.010, 0.019)	0.012 (−0.026, 0.049)	0.011 (−0.025, 0.047)	0.021 (−0.047, 0.089)
IMP, Ω	–8.50 ± 15.85	–0.052 (−1.677, 1.574)	–0.055 (−1.780, 1.671)	–0.142 (−4.615, 4.331)	–0.136 (−4.440, 4.167)	–0.257 (−8.347, 7.834)
Left Arm						
FATP, %	0.47 ± 2.21	0.192 (−0.042, 0.425)	0.203 (−0.044, 0.451)	0.527 (−0.115, 1.169)	0.507 (−0.110, 1.125)	0.954 (−0.207, 2.115)
FATM, kg	0.04 ± 0.10	0.007 (−0.003, 0.017)	0.007 (−0.004, 0.018)	0.019 (−0.010, 0.048)	0.018 (−0.009, 0.046)	0.034 (−0.017, 0.086)
FFM, kg	0.09 ± 0.16	–0.007 (−0.023, 0.010)	–0.007 (−0.024, 0.010)	–0.018 (−0.063, 0.027)	–0.017 (−0.060, 0.026)	–0.032 (−0.113, 0.049)
PMM, kg	0.08 ± 0.15	–0.007 (−0.023, 0.009)	–0.007 (−0.024, 0.010)	–0.019 (−0.062, 0.025)	–0.018 (−0.060, 0.024)	–0.034 (−0.113, 0.045)
IMP, Ω	–9.98 ± 18.71	0.344 (−1.591, 2.280)	0.365 (−1.689, 2.420)	0.947 (−4.379, 6.273)	0.911 (−4.213, 6.036)	1.714 (−7.920, 11.347)
Trunk						
FATP, %	–0.35 ± 2.76	0.246 (−0.037, 0.530)	0.262 (−0.040, 0.563)	0.678 (−0.103, 1.459)	0.652 (−0.099, 1.404)	1.227 (−0.186, 2.639)
FATM, kg	–0.48 ± 0.97	0.097 (−0.004, 0.199)	0.103 (−0.005, 0.211)	0.268 (−0.012, 0.547)	0.258 (−0.012, 0.527)	0.484 (−0.022, 0.990)
FFM, kg	–0.59 ± 1.61	–0.140 (−0.306, 0.025)	–0.149 (−0.325, 0.027)	–0.386 (−0.842, 0.070)	–0.371 (−0.810, 0.067)	–0.698 (−1.523, 0.127)
PMM, kg	–0.55 ± 1.53	–0.135 (−0.293, 0.023)	–0.143 (−0.311, 0.024)	–0.372 (−0.805, 0.062)	–0.357 (−0.775, 0.060)	–0.672 (−1.457, 0.113)
IMP, Ω	–39.14 ± 24.37	–0.228 (−2.742, 2.287)	–0.242 (−2.911, 2.428)	–0.627 (−7.547, 6.293)	–0.603 (−7.261, 6.055)	–1.134 (−13.651, 11.383)
						
phase angle, φ	–0.07 ± 0.85	–0.064 (−0.154, 0.026)	–0.068 (−0.164, 0.027)	–0.177 (−0.425, 0.071)	–0.170 (−0.409, 0.068)	–0.320 (−0.769, 0.129)
physique rating, point	1.60 ± 5.36	-0.620 (−1.182, −0.057)*	-0.658 (−1.255, −0.061)*	-1.706 (−3.254, −0.158)*	-1.641 (−3.131, −0.152)*	-3.086 (−5.886, −0.286)*

a *p < 0.05. Values in boldface are deemed statistically significant. Definitions of abbreviations: TBW, total body water; ECW, extracellular water; ICW, intracellular water; BMR, basal metabolic rate; METAAGE, metabolic age; FATP, fat percent; FATM, fat mass; FFM, fat free mass; PMM, predicted muscle mass; IMP, impedance; CI, confidence interval; PM_2.5_, particulate matter (PM) with an aerodynamic diameter of ≤2.5 μm; SD, standard deviation. Adjusted for age, sex, and body-mass index.

First, we observed a reduction in overnight total body water (TBW), extracellular water (ECW), and intracellular water (ICW). This may be explained by the excessive micturition or sweating in OSA.^7^ We found that the study participants who lived in a more polluted area had higher overnight fat mass changes and fat percentage changes of the right arm. Previous findings found that PM_2.5_ exposure was positively associated with body fat percentage.^8^ Exposure to PM_2.5_ could induce dysfunction of brown adipocytes, thus resulting in peripheral inflammation.^9^ Therefore, our findings suggest a significant association of PM_2.5_ with increases in the fat deposition of the right arm.

We observed PM_2.5_ was associated with a decrease in physique rating, which also signified a reduced level of muscle mass and an increased level of body fat.^10^ Previous findings showed that a 1.4 μg/m^3^ increase in PM_2.5_ could lead to a 0.4 kg decrease in muscle mass (*p* < 0.05) ^1^. Together, our results demonstrated that PM_2.5_ was associated with decreases in the muscle mass and increases in total body fat.

The association of particle deposition in lung regions with overnight changes in the fat mass and fat percentage of the right arm, physique rating, and visceral fat level were observed. Importantly, the absolute values of β coefficients decreased as follows: alveolar region > head and nasal region > tracheobronchial region > total lung region (all *p* < 0.05). This finding suggests that PM deposited in the lung alveoli was associated with higher increases in fat deposition than PM deposited in other lung regions. This deposited PM fraction in the alveolar region can pose a serious threat to human health. It was reported that inhaled PM_2.5_ could penetrate into the alveolar tissue and even enter the blood circulation through the air–blood barrier.^11−13^ PM_2.5_ chemical components (i.e., transition-metal elements and water-soluble ions) may translocate beyond the alveoli, causing oxidative-stress-induced adipose inflammation.^14−16^ PM_2.5_ may also induce the infiltration of macrophages and unfolded protein response in adipose tissue.^17^ More importantly, this could trigger lipogenesis, resulting in lipid deposition in adipocytes.^17^ Together, the associations of particle deposition in lung regions with the fat accumulation in various body regions were observed.

One of the limitations of this study was its relatively small sample size. Because the exposure and the outcome variables were simultaneously examined at a single time point in this cross-sectional study, the causal relationship between the exposure and the outcome could not be inferred. Therefore, a cohort study should be conducted in the future. Furthermore, we did not collect the humidity and temperature data in this study. Future works should take into consideration the cofactors of body compositions (i.e., food ingestion, hydration, alcohol, smoking, physical activities, sleep stages), relative humidity, and ambient temperature.

In this study, we examined the associations between the estimated PM deposition and overnight changes of body composition in OSA. We observed that an increase in PM deposition in lung regions, especially in the alveolar region, was associated with nocturnal changes in the fat deposition of the right arm, physique rating, and visceral fat level. Our findings demonstrated PM deposition in the alveolar region could accelerate the body fat accumulation in OSA.

## Supporting Information Available

Associations (after adjustment for nitrogen dioxide) of an interquartile range (IQR) increase in 1 year average concentrations of PM_2.5_ and associations (β coefficient) of 1 year average concentrations of PM deposition in various lung regions (i.e., total lung, head and nasal, tracheobronchial, and alveolar regions) with changes in body composition parameters in 185 obstructive sleep apnea patients using the two-pollutant model. and associations (after adjustment for ozone) of an interquartile range (IQR) increase in 1 year average concentrations of PM_2.5_ and associations (β coefficient) of 1 year average concentrations of PM deposition in various lung regions (i.e., total lung, head and nasal, tracheobronchial, and alveolar regiona) with changes in body composition parameters in 185 obstructive sleep apnea patients using the two-pollutant model (PDF)
