# Supplementary material for: Higher Particulate Matter Deposition in Alveolar Region Could Accelerate Body Fat Accumulation in Obstructive Sleep Apnea
Source: ACS Environ Au. 2022 Sep 23;3(1):12–7. doi: 10.1021/acsenvironau.2c00034 (PMC10125288; doi:10.1021/acsenvironau.2c00034)
Supplement: Supplementary file 1 — vg2c00034_si_001.pdf [file vg2c00034_si_001.pdf]

Higher particulate matter deposition in alveolar region could accelerate body fat accumulation in obstructive sleep apnea

Nguyen Thanh Tung[1,2], Shang-Yang Lin[3], Wen-Te Liu[3,10,11], Yi-Chun Kuan[4,5,6], Chih-Da Wu[7,8], Huynh Nguyen Xuan Thao[9], Hoang Ba Dung[2], Tran Phan Chung Thuy[9], Hsiao-Chi Chuang[10,11,12*]

[1]International Ph.D. Program in Medicine, College of Medicine, Taipei Medical University, Taipei, Taiwan

[2]Otorhinolaryngology Department, Cho Ray Hospital, Ho Chi Minh City, Vietnam

[3]Sleep Center, Shuang Ho Hospital, Taipei Medical University, New Taipei City, Taiwan

[4]Taipei Neuroscience Institute, Taipei Medical University, Taipei, Taiwan

[5]Department of Neurology, Taipei Medical University Shuang Ho Hospital, New Taipei City, Taiwan

[6]Department of Neurology, School of Medicine, College of Medicine, Taipei Medical University, Taipei, Taiwan

[7]Department of Geomatics, National Cheng Kung University, Tainan, Taiwan

[8]National Institute of Environmental Health Sciences, National Health Research Institutes, Miaoli, Taiwan

[9]Otorhinolaryngology Department, Faculty of Medicine, Vietnam National University Ho Chi Minh City, Ho Chi Minh City, Vietnam

[10]School of Respiratory Therapy, College of Medicine, Taipei Medical University, Taipei, Taiwan

[11]Division of Pulmonary Medicine, Department of Internal Medicine, Shuang Ho Hospital, Taipei Medical University, New Taipei City, Taiwan

[12]Cell Physiology and Molecular Image Research Center, Wan Fang Hospital, Taipei Medical University, Taipei, Taiwan

Table S1. Associations (after adjustment for nitrogen dioxide) of an interquartile range (IQR) increase in 1-year average concentrations of $PM_{2.5}$ and associations (β coefficient) of 1-year average concentrations of PM deposition in various lung regions (i.e., total lung, head and nasal, tracheobronchial, and alveolar region) with changes in body composition parameters in 185 obstructive sleep apnea patients using the two-pollutant model. Values in bold characters are deemed statistically significant (* p<0.05).

	**1-year $PM_{2.5}$**	**PM deposition in lung regions**			
		**Total lung**	**Head and nasal**	**Tracheobronchial**	**Alveolar**
	**IQR*β coefficient (CI 95%)**	**β coefficient (CI 95%)**	**β coefficient (CI 95%)**	**β coefficient (CI 95%)**	**β coefficient (CI 95%)**
**Fat percent, %**	0.152 (-0.094, 0.398)	0.162 (-0.099, 0.423)	0.419 (-0.258, 1.097)	0.403 (-0.248, 1.055)	0.759 (-0.466, 1.984)
**Fat mass, kg**	0.140 (-0.065, 0.345)	0.149 (-0.069, 0.366)	0.386 (-0.178, 0.949)	0.371 (-0.171, 0.913)	0.697 (-0.322, 1.717)
**Muscle mass, kg**	-0.096 (-0.288, 0.095)	-0.102 (-0.305, 0.101)	-0.265 (-0.791, 0.261)	-0.255 (-0.761, 0.251)	-0.479 (-1.431, 0.472)
**Visceral fat level, kg**	0.062 (-0.004, 0.128)	0.066 (-0.004, 0.136)	0.170 (-0.011, 0.352)	0.164 (-0.011, 0.338)	0.308 (-0.021, 0.636)
**Bone mass, kg**	-0.005 (-0.017, 0.006)	-0.006 (-0.018, 0.006)	-0.015 (-0.046, 0.017)	-0.014 (-0.044, 0.016)	-0.026 (-0.083, 0.030)
**Fat free mass, kg**	-0.102 (-0.303, 0.100)	-0.108 (-0.322, 0.106)	-0.280 (-0.834, 0.275)	-0.269 (-0.802, 0.264)	-0.506 (-1.508, 0.497)
**Body water**					
TBW, kg	-0.079 (-0.290, 0.133)	-0.083 (-0.308, 0.141)	-0.216 (-0.798, 0.366)	-0.208 (-0.768, 0.352)	-0.391 (-1.444, 0.662)
ECW, kg	-0.025 (-0.082, 0.031)	-0.027 (-0.087, 0.033)	-0.070 (-0.226, 0.086)	-0.067 (-0.217, 0.083)	-0.126 (-0.409, 0.156)
ICW, kg	-0.053 (-0.212, 0.105)	-0.056 (-0.225, 0.112)	-0.146 (-0.582, 0.289)	-0.141 (-0.560, 0.278)	-0.265 (-1.053, 0.523)
**Metabolism**					
BMR, kJ	-11.940 (-35.210, 11.330)	-12.676 (-37.378, 12.027)	-32.857 (-96.891, 31.176)	-31.613 (-93.223, 29.996)	-59.435 (-175.263, 56.394)
METAAGE, year	-0.225 (-0.584, 0.134)	-0.239 (-0.620, 0.143)	-0.618 (-1.607, 0.370)	-0.595 (-1.546, 0.356)	-1.119 (-2.906, 0.669)
**Right leg**					
FATP, %	0.115 (-0.068, 0.299)	0.123 (-0.072, 0.317)	0.318 (-0.186, 0.822)	0.306 (-0.179, 0.791)	0.575 (-0.337, 1.486)
FATM, kg	0.017 (-0.028, 0.063)	0.018 (-0.030, 0.067)	0.047 (-0.078, 0.173)	0.046 (-0.075, 0.166)	0.086 (-0.141, 0.313)
FFM, kg	0.003 (-0.030, 0.036)	0.003 (-0.032, 0.038)	0.009 (-0.082, 0.099)	0.008 (-0.079, 0.096)	0.016 (-0.149, 0.180)
PMM, kg	0.003 (-0.029, 0.035)	0.003 (-0.031, 0.038)	0.009 (-0.079, 0.097)	0.009 (-0.076, 0.094)	0.016 (-0.143, 0.176)
IMP, Ω	0.117 (-1.110, 1.345)	0.124 (-1.179, 1.427)	0.323 (-3.055, 3.700)	0.310 (-2.939, 3.560)	0.584 (-5.526, 6.693)
**Left leg**					
FATP, %	0.145 (-0.057, 0.346)	0.153 (-0.060, 0.367)	0.398 (-0.156, 0.952)	0.383 (-0.150, 0.916)	0.720 (-0.282, 1.722)
FATM, kg	0.023 (-0.020, 0.066)	0.024 (-0.021, 0.070)	0.063 (-0.055, 0.180)	0.060 (-0.052, 0.173)	0.114 (-0.099, 0.326)

FFM, kg	0.008 (-0.024, 0.040)	0.008 (-0.025, 0.042)	0.022 (-0.066, 0.109)	0.021 (-0.063, 0.105)	0.039 (-0.119, 0.197)
PMM, kg	0.012 (-0.018, 0.042)	0.013 (-0.019, 0.045)	0.033 (-0.050, 0.116)	0.032 (-0.049, 0.112)	0.060 (-0.091, 0.211)
IMP, Ω	-0.220 (-1.523, 1.084)	-0.233 (-1.617, 1.151)	-0.604 (-4.191, 2.983)	-0.581 (-4.033, 2.870)	-1.093 (-7.582, 5.395)
**Right arm**					
FATP, %	0.179 (-0.044, 0.401)	0.190 (-0.047, 0.426)	0.492 (-0.121, 1.105)	0.473 (-0.116, 1.063)	0.890 (-0.219, 1.998)
FATM, kg	0.011 (-0.001, 0.023)	0.011 (-0.001, 0.024)	0.030 (-0.004, 0.063)	0.029 (-0.004, 0.061)	0.054 (-0.007, 0.114)
FFM, kg	0.005 (-0.010, 0.021)	0.006 (-0.011, 0.022)	0.014 (-0.028, 0.057)	0.014 (-0.027, 0.055)	0.026 (-0.051, 0.103)
PMM, kg	0.005 (-0.010, 0.020)	0.005 (-0.011, 0.022)	0.014 (-0.029, 0.056)	0.013 (-0.028, 0.054)	0.025 (-0.052, 0.101)
IMP, Ω	-0.168 (-1.990, 1.654)	-0.178 (-2.112, 1.755)	-0.463 (-5.476, 4.551)	-0.445 (-5.268, 4.378)	-0.837 (-9.905, 8.231)
**Left arm**					
FATP, %	0.147 (-0.114, 0.408)	0.156 (-0.121, 0.434)	0.405 (-0.313, 1.124)	0.390 (-0.301, 1.081)	0.733 (-0.566, 2.033)
FATM, kg	0.007 (-0.004, 0.019)	0.008 (-0.005, 0.020)	0.020 (-0.012, 0.052)	0.019 (-0.012, 0.050)	0.036 (-0.022, 0.094)
FFM, kg	-0.003 (-0.021, 0.015)	-0.003 (-0.022, 0.016)	-0.008 (-0.058, 0.042)	-0.008 (-0.056, 0.040)	-0.014 (-0.105, 0.076)
PMM, kg	-0.003 (-0.021, 0.014)	-0.003 (-0.022, 0.015)	-0.009 (-0.058, 0.040)	-0.009 (-0.056, 0.038)	-0.016 (-0.105, 0.072)
IMP, Ω	-0.420 (-2.575, 1.735)	-0.446 (-2.734, 1.841)	-1.157 (-7.087, 4.773)	-1.113 (-6.818, 4.593)	-2.092 (-12.819, 8.634)
**Trunk**					
FATP, %	0.196 (-0.122, 0.514)	0.208 (-0.129, 0.545)	0.539 (-0.335, 1.413)	0.519 (-0.323, 1.360)	0.975 (-0.606, 2.556)
FATM, kg	0.082 (-0.032, 0.196)	0.087 (-0.034, 0.208)	0.226 (-0.087, 0.539)	0.217 (-0.084, 0.519)	0.408 (-0.158, 0.975)
FFM, kg	-0.115 (-0.301, 0.071)	-0.122 (-0.319, 0.075)	-0.316 (-0.827, 0.194)	-0.304 (-0.796, 0.187)	-0.572 (-1.496, 0.352)
PMM, kg	-0.113 (-0.290, 0.063)	-0.120 (-0.308, 0.067)	-0.312 (-0.798, 0.174)	-0.300 (-0.768, 0.168)	-0.564 (-1.443, 0.315)
IMP, Ω	-1.035 (-3.842, 1.771)	-1.099 (-4.078, 1.880)	-2.849 (-10.571, 4.874)	-2.741 (-10.171, 4.689)	-5.153 (-19.122, 8.816)
**Phase angle, φ**	-0.053 (-0.154, 0.048)	-0.056 (-0.163, 0.051)	-0.145 (-0.423, 0.133)	-0.140 (-0.407, 0.128)	-0.263 (-0.766, 0.240)
**Physique rating**	-0.421 (-1.048, 0.207)	-0.446 (-1.112, 0.219)	-1.157 (-2.883, 0.569)	-1.114 (-2.774, 0.547)	-2.094 (-5.215, 1.028)

Adjusted for age, sex, and body-mass index.

Definitions of abbreviations: TBW, total body water; ECW, extracellular water; ICW, intracellular water; BMR, basal metabolic rate; METAAGE, metabolic age; FATP, fat percent; FATM, fat mass; FFM, fat free mass; PMM, predicted muscle mass; IMP, impedance; CI, confidence interval; $PM_{2.5}$, particulate matter (PM) with an aerodynamic diameter of ≤2.5 μm; SD, standard deviation.

Table S2. Associations (after adjustment for ozone) of an interquartile range (IQR) increase in 1-year average concentrations of $PM_{2.5}$ and associations (β coefficient) of 1-year average concentrations of PM deposition in various lung regions (i.e., total lung, head and nasal, tracheobronchial, and alveolar region) with changes in body composition parameters in 185 obstructive sleep apnea patients using the two-pollutant model. Values in bold characters are deemed statistically significant (* p<0.05).

	**1-year $PM_{2.5}$**	**PM deposition in lung regions**			
		**Total lung**	**Head and nasal**	**Tracheobronchial**	**Alveolar**
	**IQR*β coefficient (CI 95%)**	**β coefficient (CI 95%)**	**β coefficient (CI 95%)**	**β coefficient (CI 95%)**	**β coefficient (CI 95%)**
**Fat percent, %**	0.184 (-0.079, 0.446)	0.195 (-0.083, 0.474)	0.506 (-0.216, 1.229)	0.487 (-0.208, 1.182)	0.915 (-0.391, 2.222)
**Fat mass, kg**	0.170 (-0.048, 0.388)	0.181 (-0.051, 0.412)	0.468 (-0.132, 1.069)	0.451 (-0.127, 1.028)	0.847 (-0.239, 1.933)
**Muscle mass, kg**	-0.117 (-0.321, 0.087)	-0.124 (-0.341, 0.092)	-0.322 (-0.884, 0.239)	-0.310 (-0.850, 0.230)	-0.583 (-1.599, 0.432)
**Visceral fat level, kg**	**0.071 (0.001, 0.142)***	**0.076 (0.001, 0.150)***	**0.196 (0.003, 0.390)***	**0.189 (0.003, 0.375)***	**0.355 (0.006, 0.705)***
**Bone mass, kg**	-0.010 (-0.023, 0.002)	-0.011 (-0.024, 0.002)	-0.029 (-0.062, 0.004)	-0.028 (-0.060, 0.004)	-0.052 (-0.112, 0.008)
**Fat free mass, kg**	-0.128 (-0.343, 0.087)	-0.136 (-0.364, 0.093)	-0.351 (-0.943, 0.240)	-0.338 (-0.907, 0.231)	-0.635 (-1.705, 0.434)
**Body water**					
TBW, kg	-0.093 (-0.319, 0.132)	-0.099 (-0.338, 0.14)	-0.256 (-0.877, 0.364)	-0.247 (-0.844, 0.350)	-0.464 (-1.586, 0.658)
ECW, kg	-0.028 (-0.089, 0.032)	-0.030 (-0.094, 0.034)	-0.077 (-0.244, 0.089)	-0.074 (-0.234, 0.086)	-0.140 (-0.441, 0.162)
ICW, kg	-0.065 (-0.234, 0.104)	-0.069 (-0.248, 0.110)	-0.179 (-0.644, 0.285)	-0.172 (-0.619, 0.274)	-0.324 (-1.165, 0.516)
**Metabolism**					
BMR, kJ	-14.838 (-39.665, 9.990)	-15.751 (-42.108, 10.605)	-40.831 (-109.151, 27.489)	-39.285 (-105.018, 26.448)	-73.857 (-197.438, 49.724)
METAAGE, year	-0.274 (-0.656, 0.108)	-0.291 (-0.697, 0.115)	-0.755 (-1.806, 0.297)	-0.726 (-1.738, 0.286)	-1.365 (-3.267, 0.538)
**Right leg**					
FATP, %	0.112 (-0.084, 0.307)	0.118 (-0.089, 0.326)	0.307 (-0.230, 0.844)	0.295 (-0.221, 0.812)	0.555 (-0.416, 1.526)
FATM, kg	0.024 (-0.024, 0.073)	0.026 (-0.026, 0.077)	0.067 (-0.067, 0.201)	0.064 (-0.064, 0.193)	0.121 (-0.121, 0.363)
FFM, kg	0.020 (-0.014, 0.055)	0.022 (-0.015, 0.058)	0.056 (-0.039, 0.151)	0.054 (-0.037, 0.146)	0.102 (-0.070, 0.274)
PMM, kg	0.021 (-0.012, 0.055)	0.022 (-0.013, 0.058)	0.058 (-0.034, 0.151)	0.056 (-0.033, 0.145)	0.105 (-0.062, 0.272)
IMP, Ω	-0.235 (-1.538, 1.068)	-0.249 (-1.632, 1.134)	-0.646 (-4.231, 2.940)	-0.621 (-4.071, 2.828)	-1.168 (-7.653, 5.317)
**Left leg**					
FATP, %	0.146 (-0.069, 0.360)	0.155 (-0.073, 0.382)	0.401 (-0.189, 0.991)	0.386 (-0.182, 0.953)	0.725 (-0.342, 1.793)
FATM, kg	0.028 (-0.017, 0.073)	0.030 (-0.019, 0.078)	0.077 (-0.048, 0.202)	0.074 (-0.046, 0.194)	0.139 (-0.087, 0.366)

FFM, kg	0.019 (-0.014, 0.053)	0.021 (-0.015, 0.056)	0.053 (-0.039, 0.146)	0.051 (-0.038, 0.141)	0.097 (-0.071, 0.264)
PMM, kg	0.023 (-0.010, 0.055)	0.024 (-0.010, 0.058)	0.062 (-0.026, 0.150)	0.060 (-0.025, 0.144)	0.112 (-0.048, 0.272)
IMP, Ω	-0.564 (-1.948, 0.820)	-0.599 (-2.068, 0.870)	-1.552 (-5.360, 2.255)	-1.493 (-5.157, 2.170)	-2.808 (-9.695, 4.079)
**Right arm**					
FATP, %	0.223 (-0.014, 0.461)	0.237 (-0.015, 0.489)	0.615 (-0.038, 1.268)	0.592 (-0.037, 1.220)	1.112 (-0.069, 2.293)
FATM, kg	0.011 (-0.002, 0.024)	0.012 (-0.002, 0.025)	0.030 (-0.006, 0.066)	0.029 (-0.005, 0.063)	0.055 (-0.010, 0.119)
FFM, kg	0.001 (-0.015, 0.018)	0.001 (-0.016, 0.019)	0.003 (-0.042, 0.049)	0.003 (-0.040, 0.047)	0.006 (-0.076, 0.088)
PMM, kg	0.001 (-0.015, 0.018)	0.001 (-0.016, 0.019)	0.004 (-0.041, 0.049)	0.004 (-0.040, 0.047)	0.007 (-0.075, 0.088)
IMP, Ω	0.187 (-1.752, 2.126)	0.199 (-1.860, 2.257)	0.515 (-4.822, 5.852)	0.495 (-4.639, 5.630)	0.931 (-8.722, 10.585)
**Left arm**					
FATP, %	0.212 (-0.067, 0.490)	0.225 (-0.071, 0.520)	0.583 (-0.183, 1.349)	0.561 (-0.176, 1.298)	1.055 (-0.331, 2.440)
FATM, kg	0.007 (-0.005, 0.020)	0.008 (-0.005, 0.021)	0.020 (-0.014, 0.055)	0.020 (-0.013, 0.052)	0.037 (-0.025, 0.099)
FFM, kg	-0.007 (-0.026, 0.013)	-0.007 (-0.028, 0.013)	-0.019 (-0.072, 0.035)	-0.018 (-0.069, 0.033)	-0.034 (-0.130, 0.063)
PMM, kg	-0.007 (-0.026, 0.012)	-0.007 (-0.028, 0.013)	-0.019 (-0.072, 0.033)	-0.019 (-0.069, 0.032)	-0.035 (-0.129, 0.059)
IMP, Ω	0.203 (-2.107, 2.513)	0.216 (-2.237, 2.668)	0.559 (-5.798, 6.916)	0.538 (-5.579, 6.654)	1.011 (-10.488, 12.510)
**Trunk**					
FATP, %	0.263 (-0.075, 0.602)	0.280 (-0.080, 0.639)	0.725 (-0.207, 1.657)	0.697 (-0.200, 1.594)	1.311 (-0.375, 2.997)
FATM, kg	0.099 (-0.022, 0.221)	0.106 (-0.023, 0.234)	0.274 (-0.060, 0.608)	0.263 (-0.058, 0.585)	0.495 (-0.109, 1.099)
FFM, kg	-0.162 (-0.360, 0.036)	-0.172 (-0.382, 0.038)	-0.446 (-0.990, 0.099)	-0.429 (-0.952, 0.095)	-0.806 (-1.790, 0.178)
PMM, kg	-0.155 (-0.343, 0.033)	-0.165 (-0.364, 0.035)	-0.427 (-0.945, 0.091)	-0.411 (-0.909, 0.088)	-0.772 (-1.709, 0.165)
IMP, Ω	-1.262 (-4.251, 1.727)	-1.340 (-4.512, 1.833)	-3.473 (-11.697, 4.752)	-3.341 (-11.254, 4.572)	-6.281 (-21.159, 8.596)
**Phase angle, φ**	-0.081 (-0.189, 0.026)	-0.086 (-0.200, 0.028)	-0.224 (-0.520, 0.072)	-0.215 (-0.500, 0.069)	-0.405 (-0.940, 0.131)
**Physique rating**	-0.457 (-1.127, 0.214)	-0.485 (-1.196, 0.227)	-1.256 (-3.100, 0.588)	-1.209 (-2.983, 0.565)	-2.273 (-5.608, 1.063)

Adjusted for age, sex, and body-mass index.

Definitions of abbreviations: TBW, total body water; ECW, extracellular water; ICW, intracellular water; BMR, basal metabolic rate; METAAGE, metabolic age; FATP, fat percent; FATM, fat mass; FFM, fat free mass; PMM, predicted muscle mass; IMP, impedance; CI, confidence interval; $PM_{2.5}$, particulate matter (PM) with an aerodynamic diameter of ≤2.5 μm; SD, standard deviation.
